# Experimental Study of the Impact of Trace Amounts of Acetylene and Methylacetylene on the Synthesis, Mechanical and Thermal Properties of Polypropylene

**DOI:** 10.3390/ijms232012148

**Published:** 2022-10-12

**Authors:** Joaquín Hernández-Fernández, Ricardo Vivas-Reyes, Carlos A. T. Toloza

**Affiliations:** 1 Chemistry Program, Department of Natural and Exact Sciences, San Pablo Campus, University of Cartagena, Cartagena 30015, Colombia; 2 Chemical Engineering Program, School of Engineering, Universidad Tecnológica de Bolivar, Parque Industrial y Tecnológico Carlos Vélez Pombo, Km 1 Vía Turbaco, Turbaco 130001, Colombia; 3 Grupo de Química Cuántica y Teórica, Facultad de Ciencias Exactas y Naturales, University of Cartagena, Cartagena 130015, Colombia; 4 Department of Natural and Exact Science, Universidad de la Costa, Barranquilla 30300, Colombia

**Keywords:** acetylene, methylacetylene, Ziegler Natta, polypropylene, catalysts, degradation

## Abstract

During the production of polymer-grade propylene, different processes are used to purify this compound and ensure that it is of the highest quality. However, some impurities such as acetylene and methyl acetylene are difficult to remove, and some of these impurities may be present in the propylene used to obtain polypropylene, which may have repercussions on the process. This study evaluates the impact of these acetylene and methyl acetylene impurities on the productivity of the polypropylene synthesis process and on the mechanical and thermal properties of the material obtained through the synthesis of eight samples with different concentrations of acetylene and eight samples with different concentrations of acetylene. We discovered that for the first concentrations of both acetylene (2 and 3 ppm) and methyl acetylene (0.03 and 0.1), the MFI, thermal recording, and mechanical properties of the resin were unaffected by the variation of the fluidity index, thermal degradation by TGA, and mechanical properties such as resistance to tension, bending, and impact. However, when the concentration exceeded 14 ppm for methyl acetylene and 12 ppm for acetylene, the resistance of this resin began to decrease linearly. Regarding production, this was affected by the first traces of acetylene and methyl acetylene progressively decreasing.

## 1. Introduction

Propylene is one of the light olefins widely used in the petrochemical industry to obtain various products such as polymers [1,2]. This generally comes from the steam cracking or catalytic cracking of naphtha [3]. This process yields high-quality propylene, but it contains cracking impurities like acetylene and methyl acetylene that must be removed [4,5]. At an industrial level, different purification techniques are used that are constantly improving to improve the amount of impurities removed from propylene, such as selective hydrogenation, which, despite being effective, does not remove 100% of the traces of acetylene and methylene present [6,7,8].

Generally, the impurities present in propylene are monitored to quantify their presence and guarantee a product with an acceptable range of them. This monitoring is mostly carried out by means of infrared spectra, tunable diode laser spectroscopy, and, in some cases, chromatography, techniques that allow the identification and quantification of the presence of these compounds [3,5,9,10,11]. Although these monitoring techniques allow the content of impurities in the polymer-grade propylene to be controlled and serve to improve purification techniques, they do not guarantee the complete removal of acetylene and methyl acetylene, allowing traces of these compounds to enter the synthesis processes in which propylene is used as the monomer [12,13].

Polypropylene is a material widely used in the manufacture of various plastic products that are part of our daily lives [14,15,16]. This resin uses propylene as its raw material for its synthesis, which requires a high degree of purity to avoid interference in the process [12,17,18]. Studies have shown that the impurities present in the propylene can affect the properties of the synthesized resin, causing the production of resin outside of specifications that becomes waste, generating more pollution, or producing resin that must be processed, generating a high consumption of energy and other raw materials to reach the desired quality [19,20].

Due to the fact that acetylene and methyl acetylene cannot be completely removed from propylene, it is necessary to study how this can affect the processes in which propylene is involved as a raw material. For this reason, in this study, the synthesis of propylene resins was carried out. We injected polypropylene with different contents of acetylene and methyl acetylene to evaluate the effects that these compounds have on the productivity of the resin synthesis, on the mechanical properties of the material, and on thermal degradation, with the aim of identifying the concentrations with which they can work without having extensive effects on the process and on the quality of the material obtained.

## 2. Results and Discussion

### 2.1. Effects of Acetylene and Methylacetylene on the Rate of Polymerization of PP

To evaluate the incidence of impurities in polypropylene, the percentages of production loss were calculated based on what was produced in the blank, as shown in Table 1. The decrease in productivity has a linear relationship inversely proportional to the concentration of acetylene or methyl acetylene present in the process. It is important to highlight that the productivity losses in the concentrations for acetylene and methyl acetylene in the eight studied concentrations of each of the compounds differ between 1 and 3%. However, for methyl acetylene, the productivity loss is evident in concentrations below 1 ppm, which indicates that there is a greater risk of affecting production with traces of methyl acetylene than with traces of acetylene. The percentage loss of productivity was calculated using Equation (1) [21,22].

% productivity loss = (1 − real produced/estimated production) × 100(1)

The productivity losses are related to the interaction that these impurities have during the synthesis, which can inhibit polymerization, preventing the desired amount from being produced, as the reactivities of methylacetylene and acetylene are higher than that of propylene when it comes to competing with the monomer for the active site of Ti, which inhibits the formation of the PP chain [23,24]. In the obtained calculations presented in Table 1, it is observable that 6% of the productivity is lost when the concentration is 0.03 ± 0.002 ppm of methyl acetylene and when the concentration is 2 ± 0.1 ppm for acetylene. At the average concentration studied (20 ppm) for each of the samples, the average productivity loss was 31% for methyl acetylene, whereas for ethylene it was 5% lower. For the highest concentration used (40 ppm), losses greater than 50% were calculated in both cases, with a difference of 2% between the compounds (56% for methylacetylene and 54% for acetylene).

### 2.2. Effects of Acetylene and Methylacetylene on Thermal Degradation

**Figure 1:**
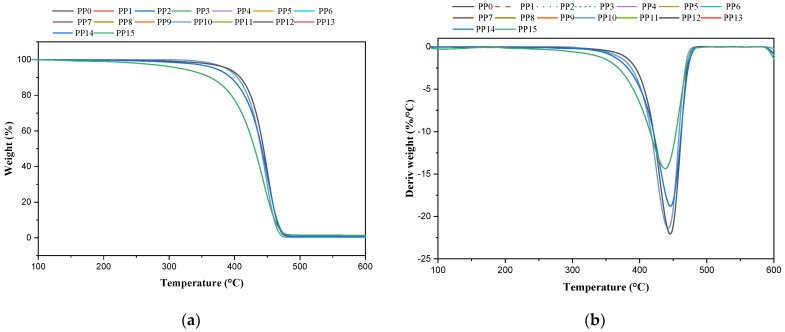
PP samples with methyl acetylene and acetylene; (**a**) TGA; (**b**) DTG.

The thermal degradation of the resins obtained with different concentrations of acetylene and methyl acetylene was measured by means of a thermogravimetric analysis (TGA, Figure 1), where two temperature ranges were evaluated; the first goes from 250 to 350 °C and the second ranges from 250 to 400 °C, where the percentage of weight lost was identified for each of the samples (Table 2). In the case of the samples with methyl acetylene, it was found that the mass loss varied by concentration groups and that for the initial concentrations (0.03 and 0.1 ppm) they did not present significant changes with respect to the blank, obtaining a mass loss equal to 1.1% for all at 350 °C and at 400 °C of 8.05%. The second group, with a mass loss equal to both 350 and 400 °C, 1.54% and 6.48%, is made up of samples PP3 and PP4, presenting a decrease in weight loss in the range of 250 to 400 °C relative to white. The third group consists of samples PP5 and PP6, which have losses of 2.3% and 11.15%, respectively, for temperature ranges of 250 to 350 °C and 250 to 400 °C. Finally, sample PP7 shows a mass loss of 5.98% from 250 to 350 °C and 20.36 for the range from 250 to 400 °C.

The samples with acetylene show the same behavior as the samples with methyl acetylene, obtaining the same mass loss in the four identified groups. The first group is made up of samples PP8, PP9, and PP10, and they present the same mass losses as samples PP0, PP1, and PP2; the second group is made up of samples PP11 and PP12, and they have the same behavior as samples PP3 and PP4; the third group is given by PP13 and PP14, and they follow the behavior of PP5 and PP6. Finally, with sample PP15, which follows the same pattern as sample PP7, it is important to highlight that for the samples with acetylene, the thermal degradation of the material is affected by concentrations greater than 3 ppm, while for methyl acetylene, it begins to be affected by concentrations greater than 0.1 ppm. For all the samples, the degradation is shown in a single step.

### 2.3. Effects of Acetylene and Methylacetylene on the MFI

**Figure 2:**
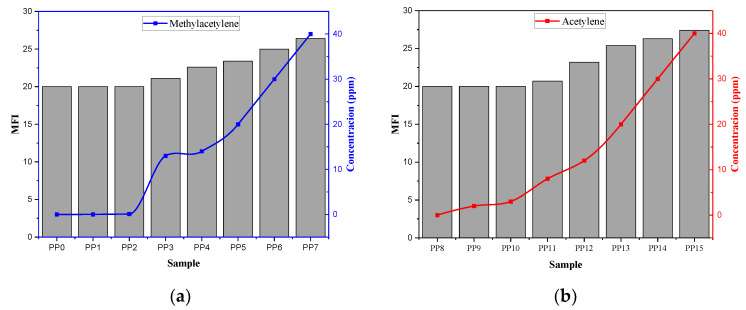
Variation of the MFI with respect to the concentration of impurities: (**a**) methyl acetylene and (**b**) acetylene.

Information on the molecular characteristics of the resins was obtained through MFI since it is strongly related to the average molecular weight. This test was performed by applying pressure for 10 min to a polymer that passes through a capillary [25]. A significant change in the MFI of a sample indicates that its molecular structure has been affected in some way by errors in the process or the presence of impurities in some of the raw materials used [26]. Figure 2 shows the variation of the MFI with respect to the concentration of acetylene and methyl acetylene in each of the samples. Initially, for concentrations below 3 ppm of methyl acetylene, there is no significant variation in the MFI, which ratifies what was found in the metric thermography study. It indicates that the resins obtained with these concentrations of impurities are not affected in any significant way at the molecular level, so their degradation would be expected to not vary. After passing through the 0.1 ppm of methyl acetylene present in the samples, an increase in the MFI is evident proportionally to the concentration of the impurity, indicating the presence of a linear relationship between the concentration of methyl acetylene and the increase in MFI. In the case of acetylene, the behavior of the MFI with respect to the concentration of the impurity follows the same pattern as explained for methyl acetylene. In this case, the variation of the MFI begins for concentrations greater than 3 ppm.

### 2.4. Effects of Acetylene and Methylacetylene on the Mechanical Properties of PP

**Figure 3:**
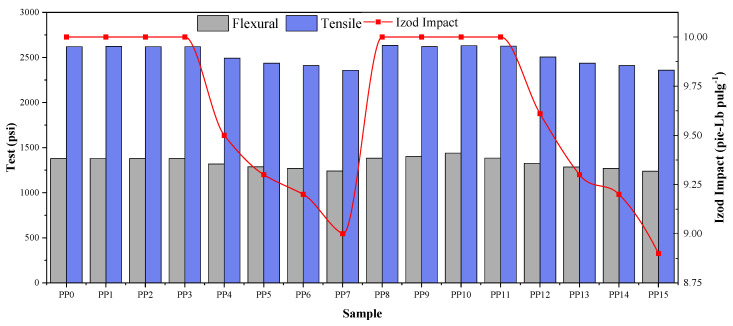
Flexural, tension, and impact tests of PP with acetylene and methyl acetylene.

The response of the polypropylene samples with acetylene and methyl acetylene to bending, tension, and impact was evaluated, as shown in Figure 3. The results obtained in these tests are related to the results obtained in the previous tests, where initially there is no significant variation in the initial concentrations to later maintain a linear relationship in the higher concentrations. The data obtained from the bending test show that for the first concentrations of methyl acetylene, the value obtained does not vary; after these concentrations, the bending values begin to gradually decrease at a mean concentration of methyl acetylene, passing the constant value of 1379 ± 32 MPa to 1319 ± 28 MPa for the 14 ppm concentration and decreasing to 1241 ± 21 MPa when it reaches a 40 ppm concentration. In contrast to this, for the samples with acetylene, there is first an increase in the first concentrations where it increases progressively from 1383 ± 36 MPa for the blank to 1439 ± 42 MPa for the concentration of 3 ppm of acetylene; later, as the concentration increases, the data obtained for bending begin to gradually decrease from 1384 ± 33 MPa obtained at the 8 ppm concentration to 1240 ± 27 MPa when the concentration reaches 40 ppm.

In the tensile test data, for the samples with methyl acetylene, the behavior does not vary with respect to the results obtained in the bending test, where a constant result is maintained in the samples PP0, PP1, PP2, and PP3 (2620 MPa), followed by a decrease in the value obtained in the following sample, presenting a result of 2492 ± 42 Mpa for sample PP4 and reaching a value of 2358 ± 31 Mpa at the highest concentration (40 ppm). Previous behavior is replicated in the samples with acetylene, being differentiated by the presence of small fluctuations of the values obtained in the first concentrations (0, 2, 3, and 8 ppm).

The behavior of the Izod impact test results replicates that explained above in the tensile test for both methyl acetylene and acetylene, wherein initially a constant value is maintained in the first four concentrations, including the 10 ft-Lb in-1 blank, followed by a constant decrease in the following concentrations until reaching 9 ft-Lb in-1. These results in the mechanical properties are related to those obtained in the MFI and in the thermogravimetric tests carried out where it is evident that in the initial concentrations there is no significant variation in structural form that could affect the properties of the material; however, as the concentration increases, an affectation can be seen in the MFI and TGA results of the samples that indicates that fewer complementary chains have been integrated into the crystal formed during polymerization, so more ends are inserted into the structural chains, decreasing the molecular weight. Thish is evidenced by the increase in the MFI and decreasing the capacity to withstand the tension, which causes failures or ruptures at lower elongation [27,28,29,30,31,32,33].

## 3. Materials and Methods

### 3.1. Reagents and Standards

For this investigation, 8 acetylene standards (0, 0.03, 0.1, 13, 14, 20, 30, and 40 ppm) and 8 methyl acetylene standards (0, 2, 3, 8, 12, 20, 30, and 40 ppm) were used). These were injected during the synthesis of PP to guarantee the presence of these compounds in the balance of propylene in the desired concentrations. Table 3 shows the other reagents used for this investigation.

### 3.2. Polymerization of Propylene

**Figure 4:**
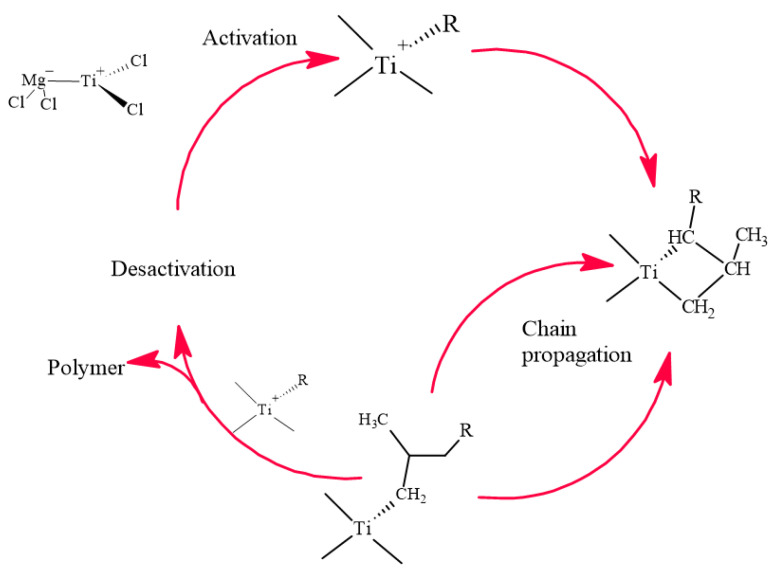
Polymerization reaction.

The polymerization was carried out in a stainless steel reactor with a capacity of 2 L, where 50 mg of the ZN catalyst was initially added and saturated with a 0.1 MPa propylene solution, with constant stirring at 300 rpm [34]. To compensate for the propylene consumed, propylene was added at 1 atm pressure continuously, controlling the flow with hydrogen after 20 min, following the polymerization mechanism shown in Figure 4. This was followed by the acetylene and methyl acetylene standards, performing a system cleaning after each polymerization to avoid contamination. Table 4 shows the amounts used for the preparation of each polypropylene sample obtained, which were evaluated for their properties.

As an additional strategy to experimentally determine whether or not acetylene and methyl acetylene reacted, complete sampling was performed at different stages of the synthesis. The absence of acetylene and methyl acetylene in stages after the polymerization stage indicates that these two impurities completely reacted inside the polymerization reactor. To analyze acetylene and methyl acetylene at all these points, the gas chromatography technique, coupled to a flame ionization detector, was be used. This will be explained in more detail in the methodology.

### 3.3. Measurement Equipment

#### 3.3.1. Analysis via Gas Chromatography with a Mass Selective Detector (GC-FID)

Quantification was performed using a gas chromatograph (Agilent 7890B, Santa Clara, CA, USA) with a front injector (250 °C, 7.88 psi, 33 mL min^−1^) and a rear injector (250 °C, 11.73 psi, 13 mL min^−1^). The volume varied between 0.25 and 1.0 mL depending on the circuit of each valve. The oven was started at 40 °C × 3 min, increased to 60 °C at 10 °C min^−1^ for four minutes, and increased again to 170 °C at 35 °C min^−1^ [35].

#### 3.3.2. Melt Flow Index (MFI)

A Tinius Olsen MP1200 plastometer was used to assess the melt flow index (MFI). According to ASTM D 1238-04c, the melt was displaced by a 2.16 kg piston at a temperature of 230 °C inside the plastometer cylinder [36,37].

#### 3.3.3. Thermogravimetric Analysis (TGA)

The samples were subjected to thermogravimetric analysis (TGA) in a TGA Q500 thermal analyzer (TA Instruments, New Castle, DE, USA) at a heating rate of 10 °C min^−1^ from 40 to 800 °C in an environment of air (50 cm^3^ min^−1^). Standard procedures were used to calibrate the instrument for weight and temperature [38].

### 3.4. Mechanical Property Tests

#### 3.4.1. Tensile Test

The capacity of composite materials to withstand axial tensile pressures and to elongate before failing is referred to as tensile strength. Both ends of the specimen are subjected to a uniaxial tensile load throughout the test. The electromechanical device stretches the material vertically after clamping the sample. On a computerized universal testing machine the tensile test was carried out using the ASTMMD638 standard (TiniusOlsen, Redhill, UK). The samples were categorized as TYPE I samples since they were created using the injection molding technique. The test sample employed in the investigation had gauge length (G), thin section width (W), and thickness (T) values of 50 mm, 12.7 mm, and 3.4 mm, respectively [39].

#### 3.4.2. Flexural Test

Using the same all-purpose testing device, the H50KL (TiniusOlsen), the flexural strength was assessed in accordance with the ASTMD790 standard. Flexural strength, or maximum stress in the outermost layer of the test specimen at fracture, is the capacity of a composite material to resist bending forces applied transversely to the axis [40].

#### 3.4.3. Impact Examination

Using the model IT504 (TiniusOlsen) pendulum impact tester, the Izod impact test was performed according to ASTMD256. One end of the notched sample was fixed by means of a cantilever vise. Each sample was assigned an AV notch of 45o and 2.5 mm depth.

## 4. Conclusions

The presence of impurities such as acetylene and methyl acetylene during the synthesis of polypropylene resins can greatly affect the production of a company, since high concentrations of these compounds are not required to reduce productivity to a little more than half, which would result in a great loss of raw materials and production. In this study, it is possible to show that the decrease in production is not only associated with visible faults such as the variation of the temperature or pressure of the process but also with the presence of impurities that, in very low concentrations, do not interfere with the properties of the resin but with the productivity of polypropylene. In general, it is evident that the properties and thermal degradation of polypropylene are affected when the concentration of methyl acetylene present in the propylene is greater than 14 ppm and the concentration of acetylene is greater than 12 ppm, which is represented by the decrease in degradation; thermal resistance of the material; resistance to tension, bending, and impact; and an increase in the flow index.

## Figures and Tables

**Table 2:** Loss of mass for acetylene and methyl acetylene samples at temperatures ranging from 250 to 350 °C and 250 to 400 °C, respectively.

Samples	% Loss at 350 °C	% Loss at 400 °C
PP0	1.1	8.05
PP1	1.1	8.05
PP2	1.1	8.05
PP3	1.5	6.48
PP4	1.5	6.48
PP5	2.3	11.15
PP6	2.3	11.15
PP7	5.9	20.36
PP8	1.1	8.05
PP9	1.1	8.05
PP10	1.1	8.05
PP11	1.5	6.48
PP12	1.5	6.48
PP13	2.3	11.15
PP14	2.3	11.15
PP15	5.9	20.36

**Table 1:** Evaluation of the productivity of the polymerization process.

Sample	Productivity ZN (TM/kg)	Methylacetylene (ppm)	Acetylene (ppm)	% Productivity Loss
PP-0	47 ± 0.1	0	0	0.00
PP-1	44 ± 0.2	0.03 ± 0.002	0	6.38
PP-2	41 ± 0.2	0.1 ± 0.02	0	11.91
PP-3	37 ± 0.3	13 ± 0.2	0	21.28
PP-4	35 ± 0.2	14 ± 0.1	0	24.89
PP-5	32 ± 0.2	20 ± 0.1	0	30.85
PP-6	29 ± 0.1	30 ± 0.1	0	38.94
PP-7	21 ± 0.1	40 ± 0.1	0	55.96
PP-8	47 ± 0.1	0	0	0.00
PP-9	44 ± 0.2	0	2 ± 0.1	6.17
PP-10	42 ± 0.2	0	3 ± 0.1	9.57
PP-11	40 ± 0.2	0	8 ± 0.1	14.89
PP-12	37 ± 0.3	0	12 ± 0.2	20.21
PP-13	35 ± 0.2	0	20 ± 0.1	26.17
PP-14	28 ± 0.2	0	30 ± 0.1	40.64
PP-15	21 ± 0.2	0	40 ± 0.1	54.47

**Table 3:** Reagents.

Materials	Used Supplier	Purity
Diisobutyl phthalate (DIBP)	(in-house donor) Sudchemie, Germany	99.99%
Triethylaluminium	(co-catalyst)	98%
	Merck, Germany	
Cyclohexyl methyl dimethoxysilane (CMDS)	(external donor) Merck, Germany	99.9%
Hydrogen	Lynde	99.999%
Nitrogen	Lynde	99.999%
Propylene	Airgas	99.999%

**Table 4:** Polymerization reagents.

Materials	Samples															
	PP0	PP1	PP2	PP3	PP4	PP5	PP6	PP7	PP8	PP9	PP10	PP11	PP12	PP13	PP14	PP15
**Catalyst, Kh/h**	5	5	5	5	5	5	5	5	5	5	5	5	5	5	5	5
**Propylene, TM/h**	1.2	1.2	1.2	1.2	1.2	1.2	1.2	1.2	1.2	1.2	1.2	1.2	1.2	1.2	1.2	1.2
**TEAl, Kg/h**	0.25	0.25	0.25	0.25	0.25	0.25	0.25	0.25	0.25	0.25	0.25	0.25	0.25	0.25	0.25	0.25
**Hydrogen, g/h**	30	30	30	30	30	30	30	30	30	30	30	30	30	30	30	30
**Acetylene (ppm)**	0	2	3	8	12	20	30	40	0	0	0	0	0	0	0	0
**Methyl** **acetylene (ppm)**	0	0	0	0	0	0	0	0	0	0.03	0.1	13	14	20	30	40
**Selectivity control agent, mol/h**	1	1	1	1	1	1	1	1	1	1	1	1	1	1	1	1
***T*, °C**	70	70	70	70	70	70	70	70	70	70	70	70	70	70	70	70
**Pressure, bar**	27	27	27	27	27	27	27	27	27	27	27	27	27	27	27	27

## Data Availability

Not applicable.
